# Hexaaqua­magnesium bis­(4-amino-3-methyl­benzene­sulfonate)

**DOI:** 10.1107/S1600536809046595

**Published:** 2009-11-11

**Authors:** Wei Zhang, Yuan-Tao Chen

**Affiliations:** a Department of Chemistry, Qinghai Normal University, Xining 810008, People’s Republic of China

## Abstract

In the title mol­ecular salt, [Mg(H_2_O)_6_](C_7_H_8_NO_3_S)_2_, the Mg^2+^ cation lies on an inversion centre. In the crystal, the components are linked by N—H⋯O and O—H⋯O hydrogen bonds, thereby generating sheets parallel to (001).

## Related literature

For the isostructural cobalt-containing compound, see: Zhang & Chen (2009 ▶).
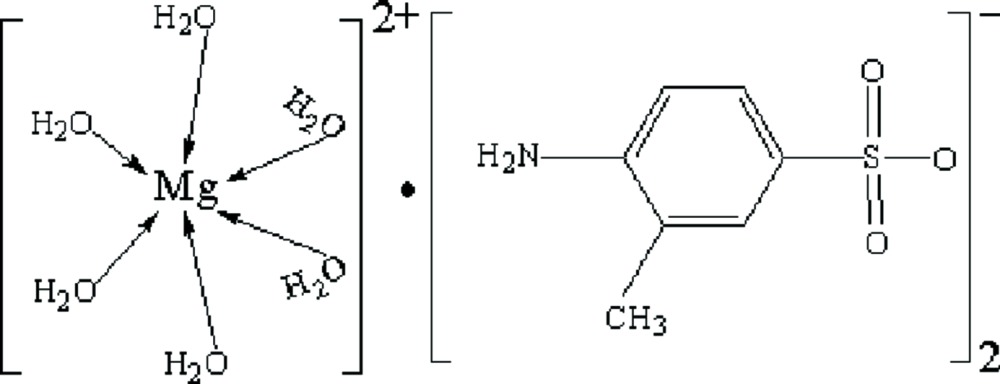


## Experimental

### 

#### Crystal data

[Mg(H_2_O)_6_](C_7_H_8_NO_3_S)_2_
*M* *_r_* = 504.81
Monoclinic, 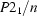

*a* = 6.3048 (13) Å
*b* = 7.0395 (15) Å
*c* = 24.356 (5) Å
β = 93.921 (3)°
*V* = 1078.5 (4) Å^3^
*Z* = 2
Mo *K*α radiation
μ = 0.34 mm^−1^
*T* = 273 K
0.23 × 0.16 × 0.12 mm

#### Data collection

Bruker SMART CCD diffractometer
Absorption correction: multi-scan (*SADABS*; Bruker, 2000 ▶) *T*_min_ = 0.926, *T*_max_ = 0.960
5398 measured reflections
1918 independent reflections
1779 reflections with *I* > 2σ(*I*)
*R*_int_ = 0.020

#### Refinement

*R*[*F* ^2^ > 2σ(*F* ^2^)] = 0.054
*wR*(*F* ^2^) = 0.138
*S* = 1.27
1918 reflections
144 parameters
9 restraints
H-atom parameters constrained
Δρ_max_ = 0.37 e Å^−3^
Δρ_min_ = −0.42 e Å^−3^

### 

Data collection: *SMART* (Bruker, 2000 ▶); cell refinement: *SAINT* (Bruker, 2000 ▶); data reduction: *SAINT*; program(s) used to solve structure: *SHELXS97* (Sheldrick, 2008 ▶); program(s) used to refine structure: *SHELXL97* (Sheldrick, 2008 ▶); molecular graphics: *SHELXTL* (Sheldrick, 2008 ▶); software used to prepare material for publication: *SHELXTL*.

## Supplementary Material

Crystal structure: contains datablocks global, I. DOI: 10.1107/S1600536809046595/hb5189sup1.cif

Structure factors: contains datablocks I. DOI: 10.1107/S1600536809046595/hb5189Isup2.hkl

Additional supplementary materials:  crystallographic information; 3D view; checkCIF report

## Figures and Tables

**Table 1:** Selected bond lengths (Å)

Mg1—O4	2.029 (3)
Mg1—O6	2.071 (3)
Mg1—O5	2.075 (3)

**Table 2:** Hydrogen-bond geometry (Å, °)

*D*—H⋯*A*	*D*—H	H⋯*A*	*D*⋯*A*	*D*—H⋯*A*
N1—H1*A*⋯O3^i^	0.86	2.48	3.208 (5)	143
N1—H1*B*⋯O6^ii^	0.86	2.54	3.133 (5)	127
O4—H7⋯O3	0.85	1.90	2.748 (4)	178
O4—H8⋯O1^iii^	0.85	1.94	2.778 (4)	169
O5—H9⋯O2^iii^	0.85	1.97	2.810 (4)	168
O5—H10⋯O1^iv^	0.85	1.95	2.790 (4)	170
O6—H11⋯O2	0.85	1.94	2.776 (4)	169
O6—H12⋯O3^v^	0.85	2.01	2.835 (4)	163

Symmetry codes: (i) 
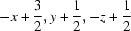
; (ii) 
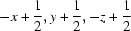
; (iii) 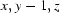; (iv) 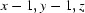; (v) 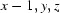.

**Fig. 1.**
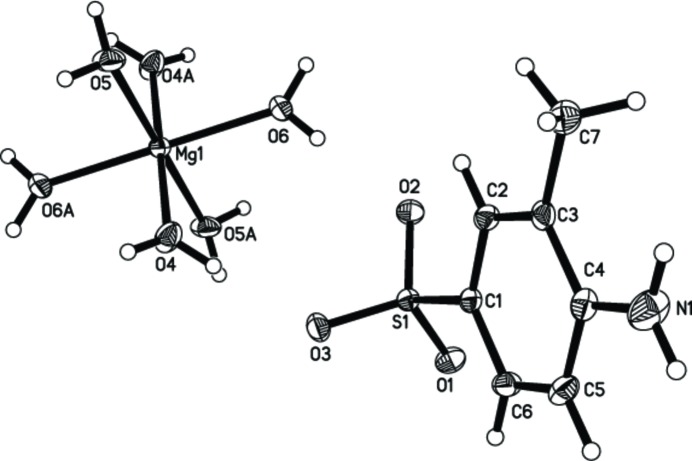
The molecular structure of (I) showing 30% displacement ellipsoids. Atoms with the suffix A are generated by (–x, –y, –z).

## supplementary crystallographic information

### Experimental

A solution of 1.0 mmol 4-amino-3-methyl-benzenesulfonic acid and 1.0 mmol NaOH in 10 ml ethanol was added to a solution of 0.5 mmol MgCl_2_6H_2_O in 5 ml ethanol at room temperature. The mixture was refluxed for 3 h with stirring, then the resulting precipitate was filtered, washed, and dried *in vacuo* over P_4_O_10_ for 48 h. Colourless blocks of (I) were obtained by slowly evaporating from ethanol at room temperature.

### Refinement

The H atoms were positioned geometrically (C—H = 0.93–0.96, N—H = 0.86, O—H = 0.85Å) and refined as riding with *U*_iso_(H)= 1.2 *U*_eq_(C,N) or 1.5 *U*_eq_(O).

### Crystal data

**Table:**

[Mg(H_2_O)_6_](C_7_H_8_NO_3_S)_2_	*F*(000) = 532
*M**_r_* = 504.81	*D*_x_ = 1.555 Mg m^−^^3^
Monoclinic, *P*2_1_/*n*	Mo *K*α radiation, λ = 0.71073 Å
Hall symbol: -P 2yn	Cell parameters from 3577 reflections
*a* = 6.3048 (13) Å	θ = 3.0–28.6°
*b* = 7.0395 (15) Å	µ = 0.34 mm^−^^1^
*c* = 24.356 (5) Å	*T* = 273 K
β = 93.921 (3)°	Block, colourless
*V* = 1078.5 (4) Å^3^	0.23 × 0.16 × 0.12 mm
*Z* = 2	

### Data collection

**Table:**

Bruker SMART CCD diffractometer	1918 independent reflections
Radiation source: fine-focus sealed tube	1779 reflections with *I* > 2σ(*I*)
graphite	*R*_int_ = 0.020
ω scans	θ_max_ = 25.1°, θ_min_ = 1.7°
Absorption correction: multi-scan (*SADABS*; Bruker, 2000)	*h* = −6→7
*T*_min_ = 0.926, *T*_max_ = 0.960	*k* = −8→8
5398 measured reflections	*l* = −28→25

### Refinement

**Table:**

Refinement on *F*^2^	Primary atom site location: structure-invariant direct methods
Least-squares matrix: full	Secondary atom site location: difference Fourier map
*R*[*F*^2^ > 2σ(*F*^2^)] = 0.054	Hydrogen site location: inferred from neighbouring sites
*wR*(*F*^2^) = 0.138	H-atom parameters constrained
*S* = 1.27	*w* = 1/[σ^2^(*F*_o_^2^) + (0.0175*P*)^2^ + 3.9593*P*] where *P* = (*F*_o_^2^ + 2*F*_c_^2^)/3
1918 reflections	(Δ/σ)_max_ < 0.001
144 parameters	Δρ_max_ = 0.37 e Å^−^^3^
9 restraints	Δρ_min_ = −0.42 e Å^−^^3^

### Special details

**Table:**

Geometry. All e.s.d.'s (except the e.s.d. in the dihedral angle between two l.s. planes) are estimated using the full covariance matrix. The cell e.s.d.'s are taken into account individually in the estimation of e.s.d.'s in distances, angles and torsion angles; correlations between e.s.d.'s in cell parameters are only used when they are defined by crystal symmetry. An approximate (isotropic) treatment of cell e.s.d.'s is used for estimating e.s.d.'s involving l.s. planes.
Refinement. Refinement of *F*^2^ against ALL reflections. The weighted *R*-factor *wR* and goodness of fit *S* are based on *F*^2^, conventional *R*-factors *R* are based on *F*, with *F* set to zero for negative *F*^2^. The threshold expression of *F*^2^ > σ(*F*^2^) is used only for calculating *R*-factors(gt) *etc*. and is not relevant to the choice of reflections for refinement. *R*-factors based on *F*^2^ are statistically about twice as large as those based on *F*, and *R*- factors based on ALL data will be even larger.

### Fractional atomic coordinates and isotropic or equivalent isotropic displacement parameters (Å^2^)

**Table:**

	*x*	*y*	*z*	*U*_iso_*/*U*_eq_	
Mg1	0.0000	0.0000	0.0000	0.0216 (4)	
S1	0.40358 (14)	0.50773 (14)	0.09733 (4)	0.0218 (3)	
O1	0.4934 (5)	0.6661 (4)	0.06800 (11)	0.0317 (7)	
O2	0.1712 (4)	0.5034 (4)	0.09088 (12)	0.0297 (7)	
O3	0.4968 (4)	0.3258 (4)	0.08251 (12)	0.0304 (7)	
O4	0.2913 (5)	0.0074 (4)	0.04083 (13)	0.0375 (8)	
H7	0.3575	0.1051	0.0533	0.056*	
H8	0.3647	−0.0883	0.0519	0.056*	
O5	−0.0976 (5)	−0.1937 (4)	0.05737 (13)	0.0366 (8)	
H9	−0.0146	−0.2881	0.0624	0.055*	
H10	−0.2222	−0.2401	0.0564	0.055*	
O6	−0.1022 (5)	0.2297 (4)	0.04427 (13)	0.0340 (7)	
H12	−0.2251	0.2721	0.0498	0.052 (16)*	
H11	−0.0147	0.3033	0.0618	0.11 (3)*	
N1	0.6584 (7)	0.6327 (6)	0.33201 (15)	0.0465 (11)	
H1A	0.7828	0.6779	0.3408	0.056*	
H1B	0.5739	0.6070	0.3573	0.056*	
C1	0.4753 (6)	0.5435 (5)	0.16775 (16)	0.0217 (8)	
C2	0.3351 (6)	0.5006 (6)	0.20762 (16)	0.0248 (8)	
H2	0.2013	0.4519	0.1971	0.030*	
C3	0.3929 (6)	0.5297 (5)	0.26277 (16)	0.0254 (9)	
C4	0.5949 (7)	0.6021 (6)	0.27807 (16)	0.0280 (9)	
C5	0.7335 (7)	0.6445 (6)	0.23738 (17)	0.0305 (9)	
H5	0.8675	0.6936	0.2474	0.037*	
C6	0.6748 (6)	0.6147 (6)	0.18287 (17)	0.0276 (9)	
H6	0.7689	0.6424	0.1562	0.033*	
C7	0.2434 (7)	0.4831 (7)	0.30672 (18)	0.0363 (10)	
H7A	0.1193	0.4209	0.2903	0.054*	
H7B	0.2022	0.5982	0.3243	0.054*	
H7C	0.3136	0.4006	0.3336	0.054*	

### Atomic displacement parameters (Å^2^)

**Table:**

	*U*^11^	*U*^22^	*U*^33^	*U*^12^	*U*^13^	*U*^23^
Mg1	0.0199 (9)	0.0211 (9)	0.0240 (9)	−0.0004 (8)	0.0032 (7)	0.0008 (8)
S1	0.0198 (5)	0.0208 (5)	0.0249 (5)	−0.0004 (4)	0.0011 (3)	−0.0008 (4)
O1	0.0331 (16)	0.0318 (16)	0.0304 (15)	−0.0064 (13)	0.0045 (12)	0.0080 (13)
O2	0.0208 (14)	0.0326 (16)	0.0351 (16)	0.0015 (13)	−0.0024 (11)	−0.0017 (13)
O3	0.0299 (16)	0.0279 (16)	0.0335 (16)	0.0043 (13)	0.0021 (12)	−0.0089 (13)
O4	0.0303 (16)	0.0275 (16)	0.0527 (19)	−0.0020 (13)	−0.0117 (14)	−0.0009 (15)
O5	0.0264 (16)	0.0361 (17)	0.0486 (19)	0.0009 (13)	0.0121 (14)	0.0161 (15)
O6	0.0273 (16)	0.0312 (16)	0.0442 (18)	0.0002 (14)	0.0083 (13)	−0.0131 (14)
N1	0.051 (2)	0.060 (3)	0.028 (2)	−0.018 (2)	−0.0021 (17)	−0.004 (2)
C1	0.0220 (19)	0.0166 (18)	0.0266 (19)	0.0008 (15)	0.0018 (15)	−0.0004 (15)
C2	0.0227 (19)	0.0198 (19)	0.032 (2)	−0.0004 (16)	0.0019 (16)	0.0006 (17)
C3	0.032 (2)	0.0158 (19)	0.029 (2)	0.0006 (16)	0.0067 (16)	0.0000 (16)
C4	0.034 (2)	0.022 (2)	0.028 (2)	−0.0019 (17)	0.0015 (17)	−0.0017 (17)
C5	0.027 (2)	0.029 (2)	0.035 (2)	−0.0064 (18)	−0.0047 (18)	−0.0018 (18)
C6	0.023 (2)	0.030 (2)	0.031 (2)	−0.0028 (17)	0.0046 (16)	0.0009 (18)
C7	0.047 (3)	0.030 (2)	0.033 (2)	−0.006 (2)	0.012 (2)	0.002 (2)

### Geometric parameters (Å, °)

**Table:**

Mg1—O4^i^	2.029 (3)	N1—C4	1.364 (5)
Mg1—O4	2.029 (3)	N1—H1A	0.8600
Mg1—O6^i^	2.071 (3)	N1—H1B	0.8600
Mg1—O6	2.071 (3)	C1—C6	1.380 (5)
Mg1—O5	2.075 (3)	C1—C2	1.390 (5)
Mg1—O5^i^	2.075 (3)	C2—C3	1.383 (6)
S1—O1	1.459 (3)	C2—H2	0.9300
S1—O2	1.463 (3)	C3—C4	1.399 (6)
S1—O3	1.465 (3)	C3—C7	1.510 (6)
S1—C1	1.762 (4)	C4—C5	1.398 (6)
O4—H7	0.8499	C5—C6	1.370 (6)
O4—H8	0.8500	C5—H5	0.9300
O5—H9	0.8500	C6—H6	0.9300
O5—H10	0.8500	C7—H7A	0.9600
O6—H12	0.8500	C7—H7B	0.9600
O6—H11	0.8500	C7—H7C	0.9600
			
O4^i^—Mg1—O4	180.0	H12—O6—H11	105.9
O4^i^—Mg1—O6^i^	91.62 (13)	C4—N1—H1A	120.0
O4—Mg1—O6^i^	88.38 (13)	C4—N1—H1B	120.0
O4^i^—Mg1—O6	88.38 (13)	H1A—N1—H1B	120.0
O4—Mg1—O6	91.62 (13)	C6—C1—C2	120.3 (4)
O6^i^—Mg1—O6	180.0	C6—C1—S1	118.7 (3)
O4^i^—Mg1—O5	90.75 (12)	C2—C1—S1	121.0 (3)
O4—Mg1—O5	89.25 (12)	C3—C2—C1	120.6 (4)
O6^i^—Mg1—O5	87.41 (12)	C3—C2—H2	119.7
O6—Mg1—O5	92.59 (12)	C1—C2—H2	119.7
O4^i^—Mg1—O5^i^	89.25 (12)	C2—C3—C4	119.2 (4)
O4—Mg1—O5^i^	90.75 (12)	C2—C3—C7	121.4 (4)
O6^i^—Mg1—O5^i^	92.59 (12)	C4—C3—C7	119.4 (4)
O6—Mg1—O5^i^	87.41 (12)	N1—C4—C5	119.5 (4)
O5—Mg1—O5^i^	180.0	N1—C4—C3	121.1 (4)
O1—S1—O2	112.49 (18)	C5—C4—C3	119.4 (4)
O1—S1—O3	111.84 (17)	C6—C5—C4	120.9 (4)
O2—S1—O3	111.78 (17)	C6—C5—H5	119.5
O1—S1—C1	106.55 (17)	C4—C5—H5	119.5
O2—S1—C1	107.23 (17)	C5—C6—C1	119.7 (4)
O3—S1—C1	106.52 (17)	C5—C6—H6	120.2
Mg1—O4—H7	127.0	C1—C6—H6	120.2
Mg1—O4—H8	126.1	C3—C7—H7A	109.5
H7—O4—H8	106.5	C3—C7—H7B	109.5
Mg1—O5—H9	113.8	H7A—C7—H7B	109.5
Mg1—O5—H10	123.4	C3—C7—H7C	109.5
H9—O5—H10	105.2	H7A—C7—H7C	109.5
Mg1—O6—H12	132.6	H7B—C7—H7C	109.5
Mg1—O6—H11	121.5		
			
O1—S1—C1—C6	−38.2 (4)	C2—C3—C4—N1	−179.9 (4)
O2—S1—C1—C6	−158.9 (3)	C7—C3—C4—N1	−0.5 (6)
O3—S1—C1—C6	81.3 (3)	C2—C3—C4—C5	0.4 (6)
O1—S1—C1—C2	142.0 (3)	C7—C3—C4—C5	179.8 (4)
O2—S1—C1—C2	21.4 (4)	N1—C4—C5—C6	179.8 (4)
O3—S1—C1—C2	−98.4 (3)	C3—C4—C5—C6	−0.5 (6)
C6—C1—C2—C3	0.4 (6)	C4—C5—C6—C1	0.6 (6)
S1—C1—C2—C3	−179.8 (3)	C2—C1—C6—C5	−0.5 (6)
C1—C2—C3—C4	−0.3 (6)	S1—C1—C6—C5	179.7 (3)
C1—C2—C3—C7	−179.7 (4)		

Symmetry codes: (i) −*x*, −*y*, −*z*.

### Hydrogen-bond geometry (Å, °)

**Table:**

*D*—H···*A*	*D*—H	H···*A*	*D*···*A*	*D*—H···*A*
N1—H1A···O3^ii^	0.86	2.48	3.208 (5)	143
N1—H1B···O6^iii^	0.86	2.54	3.133 (5)	127
O4—H7···O3	0.85	1.90	2.748 (4)	178
O4—H8···O1^iv^	0.85	1.94	2.778 (4)	169
O5—H9···O2^iv^	0.85	1.97	2.810 (4)	168
O5—H10···O1^v^	0.85	1.95	2.790 (4)	170
O6—H11···O2	0.85	1.94	2.776 (4)	169
O6—H12···O3^vi^	0.85	2.01	2.835 (4)	163

Symmetry codes: (ii) −*x*+3/2, *y*+1/2, −*z*+1/2; (iii) −*x*+1/2, *y*+1/2, −*z*+1/2; (iv) *x*, *y*−1, *z*; (v) *x*−1, *y*−1, *z*; (vi) *x*−1, *y*, *z*.
